# Electrochemical Production of Bismuth in the KCl–PbCl_2_ Melt

**DOI:** 10.3390/ma14195653

**Published:** 2021-09-28

**Authors:** Pavel Alexandrovich Arkhipov, Yury Pavlovich Zaikov, Yuliya Rinatovna Khalimullina, Stepan Pavlovich Arkhipov

**Affiliations:** Electrolysis Section, Institute of High Temperature Electrochemistry, Ural Branch, Russian Academy of Sciences, 620990 Ekatherinburg, Russia; dir@ihte.uran.ru (Y.P.Z.); yu.Halim@ihte.uran.ru (Y.R.K.); iamstepa@gmail.com (S.P.A.)

**Keywords:** molten salt, metallic alloy, anode process, Pb, Bi

## Abstract

An anode dissolution of binary metallic lead–bismuth alloys with different concentrations of components has been studied in the KCl–PbCl_2_ molten eutectic. The dissolution of lead is found to be a basic process for the alloys of Pb–Bi (59.3–40.7), Pb–Bi (32.5–67.5), Pb–Bi (7.0–93.0) compositions in the whole interval of studied anode current densities. A limiting diffusion current of lead dissolution was observed at 2 A/cm^2^ and 0.1 A/cm^2^ for the alloys of Pb–Bi (5.0–95.0) and Pb–Bi (3.0–97.0) compositions, respectively. The dissolution of bismuth takes place at the anode current densities exceeding the mentioned values. The number of electrons participating in the electrode reactions is detected for each mechanism. Based on the theoretical analysis, the experimental electrolysis of bismuth was performed in the laboratory-scale electrolytic cell with a porous ceramic diaphragm. The final product contained pure bismuth with a lead concentration of 3.5 wt.%.

## 1. Introduction

Development of the technology for the electrolytic bismuth production from fusible alloys using molten chloride systems requires understanding the mechanism of processes on the liquid-metallic electrode. Baseline information on the anode transformations of binary lead–bismuth alloys is essential for process engineering of pure metallic lead and bismuth, as well as Pb–Bi alloys of definite compositions used as candidate liquid-metallic coolants [1,2,3]. Modern international ecological and environmental management gives special emphasis to handling materials containing lead and lead alloys. The countries, including the Russian Federation [4], China [5], South Korea [6], Ghana [7], Belgium [8] and others, where industrial production of lead is well developed, are obliged to arrange the lead recycling facilities. The technology of bismuth electrorefining from fusible alloys utilizing chloride systems may be associated with lead electrorefining.

Both cathode and anode processes play key roles in the electrochemical separation of the metals; lead electrodissolution from the Pb–Bi alloys takes place at the anode, whereas lead (II) ions reduce electrolytically to metallic Pb at the cathode. Zhang et al. [9] reported on the analysis of the electrochemical reduction of lead ions in the LiC1–KC1–PbCl_2_–MgCl_2_ melt by voltammetry and chronopotentiometry. The electrochemical reaction of lead ions reduction is reversible according to the two-valence scheme; the diffusion coefficient, calculated according to Sanda’s equation, is equal to D_Pb(II)_ = 2.26 × l0^−5^ cm^2^/s for 873 K. It is found that potentiostatic lithium reduction on the preliminary deposited lead results in the formation of a liquid Li–Pb alloy, whereas Mg–Li–Pb alloys form only after an MgCl_2_ addition to the melt. Later, Han et al. [10] studied the kinetics of cathode processes on the indifferent electrode at lead ions Pb^2+^ reduction in the LiCl–KCl melt. Zhu [11] analyzed Pb (II) electrochemical behavior on the tungsten electrode in the molten NaCl–KCl eutectic at 700 °C using voltammetry and chronopotentiometry. It was found that Pb(II) reduction to metallic Pb in the NaCl–KCl melts proceeds according to the two-electron electrode mechanism. This reaction is quasi-reversible and diffusion-controlled.

Haarberg et al. [12] studied the electroreduction of lead, magnesium and zinc ions on the tungsten and glassy carbon electrodes in the KCl–LiCl melt using linear voltammetry. The calculated values of diffusion coefficients of the two-valence lead ions are described by the empirical equation D_Pb(II)_ = 5.3 × 10^3−^ ∙ exp(−35800/RT) cm^2^/s within the temperature range of 400–500 °C [12].

Electrode processes of Pb (II) ions reduction in the LiCl–KCl–PbCl_2_ electrolyte were studied at the Institute of High-Temperature Electrochemistry by voltammetry and stationary polarization [13]. Kinetic parameters of the cathode process were determined. The analysis of the stationary polarization curves as functions of the Pb^2+^ electroreduction process on temperature and concentration of the potential-determining ion in the melt reveals that the cathode process has a diffusion regime. The number of electrons in the cathode reaction was calculated for three different temperatures, and it was found that 2.00 electrons took part in the reaction at 723 K, 1.96 electrons at 773 K and 1.97 electrons at 823 K [13]. The analysis of voltammograms illustrates that the growth of the potential sweep rate towards the cathode side results in the increase in the value of the single peak along with the absolute value at a negligible shift of the peak potential. The characteristic dependence of Ip–V^1/2^ is a straight line, which extrapolation leads to the origin of the coordinates. The obtained results testify that under the experimental conditions, the process of Pb^2+^ ions reduction in the chloride eutectic is controlled by diffusion along with the whole interval of potential sweep rates. The calculated values of the Pb^2+^ ions diffusion coefficients in the LiCl–KCl–PbCl_2_ melt at 723, 773 and 823 K using the Bersin-Delahay equation are 1.91 × 10^−5^ cm^2^/s, 2.29 × 10^−5^ cm^2^/s, 2.59 × 10^−5^ cm^2^/s, respectively [13].

The review of the papers on the metallic lead production and application in ionic melts proved that the cathode process is not limited by any noticeable kinetic obstacles at 723–823 K, whereas under the diffusion regime and satisfactory rates of lead ions transfer towards the electrode at the near-equilibrium potentials.

The equilibrium potentials of the Pb–Bi [14], Pb–SbBi_i,j_ [15,16] alloys were measured in KCl–PbCl_2_ for a wide range of compositions in the temperature interval of 723–893 K. The EMF method was used to calculate the thermodynamic functions of binary and ternary Pb–Bi, Pb–SbBi_i,j_ systems. Given the classification of metallic alloys based on their thermodynamic functions proposed by Kaptay [17,18], the values of excess partial enthalpy and excess partial entropy of the pseudo-binary Pb–SbBi system are small and tend to zero.

Ebe et al. [19] report on the electrochemical transformations of Sb and Bi in the AlCl_3–_KCl–NaCl alloy with different aluminum, potassium and sodium trichlorides concentrations by the voltammetry method. The voltammograms of the Sb^2+^ containing melts demonstrate one peak both in the cathode and in the anode directions of the potential sweep. The voltammograms of the bismuth trichloride-containing electrolyte have a more complex form. The Bi(III)/Bi(I) redox reaction is suggested to proceed at the potential of 1.1 V relative to the chloride reference electrode under the conditions of the experiment [19].

The present paper reports on the new achievements in the development of the technology for electrochemical production of non-ferrous metals in chloride melts presented in previous papers [20,21,22]. The electric conductivity and liquidus temperature of the molten KCl–PbCl_2_ [20] and CsCl–PbCl_2_ [21,22] systems containing lead oxide have been measured. Three types of laboratory electrolytic cells for electrorefining of metallic Pb containing Sb and Bi have been developed and tested [23]: the electrolytic cell with a bipolar metallic electrode, the electrolytic cell with two anodes and one cathode, and that with a porous diaphragm. All construction proved to be efficient for the separation of lead and other metals. The final product, i.e., grade lead, is formed at the cathode, and Bi–Sb or Pb–Sb alloys are obtained at the anode. As opposed to the constructions with the dielectric barriers, the electrolytic cell with porous diaphragm was found to have a twice higher process rate and several times lower cell voltage [23].

This work is devoted to the analysis of the effect of the composition of liquid-metallic lead–bismuth systems containing more than 90% of Bi on the anode polarization in the molten KCl–PbCl_2_ mixture and to the development of the technology for electrolytic production of Bi from the Pb–Bi alloy.

## 2. Materials and Methods

The anode dissolution of lead–bismuth alloys and individual lead and bismuth was studied at the electric current cutoff under the galvanostatic mode using an IPC-Pro galvanostat/potentiostat (Scientific Technical Company “VOLTA”, St. Petersburg, Russia). The polarization was also measured at the electric current cutoff; the amplitude of constant current impulses varied from 0.001 to 1 A, and the polarization extended 5–7 s. The electrochemical cell, described in [13], was used for the experiments and anode polarization measuring procedures. An alundum crucible with the electrolyte, auxiliary electrode, working electrode, reference electrode and thermocouple in alundum sheath was located on the cell bottom. Experiments were performed in the sealed cell in a purified argon atmosphere; all oxygen traces were thoroughly removed. The cell was located at a resistance furnace preliminary heated to the desired temperature under the excess argon pressure. As a working electrode, i.e., an anode, we used an alloy of the required composition or elementary lead/elementary bismuth. The compositions of the auxiliary and working electrodes were the same. An equimolar potassium and lead chloride mixture served as the electrolyte both for auxiliary and for working electrodes. The measurements were performed relative to metallic lead in contact with KCl–PbCl_2_. An electrolyte permeable diaphragm separated the electrolyte between the working electrode and the reference electrode. Molybdenum rods (**⌀** 1 mm) were utilized as current leads to the liquid-metal electrodes [24,25].

The electrolyte was prepared from chemically pure PbCl_2_ and KCl reagents (“VEKTON” CJSC, St. Petersburg, Russia). The alloys were prepared from C-1 grade lead, Bi-00 grade bismuth (99.985 purity, Ural Mining Metallurgical Company, V. Pyshma, Russia). The samples of the metallic alloys and electrolyte compositions were taken before and after the experiment by the atomic-absorption method using an Optic emission spectrometer with the inductively bound plasma Perkin Elmer OPTIMA 4300 DV (PerkinElmer, Waltham, MA, USA). The compositions of the samples remained unchanged.

## 3. Results

The experimental measurements of anode polarization of metallic Pb, Bi and double Pb–Bi alloys were performed at 823 K. Table 1 illustrates compositions of the working electrodes.

To facilitate the analysis of the measurement results, the measured polarization values were recalculated relative to the Cl_2_/Cl^−^ chloride reference electrode using the equation derived in [14]:

E_Pb2+/Pb_ = 0.523 ∙ T − 1734.0 mV (1)

**Figure 1:**
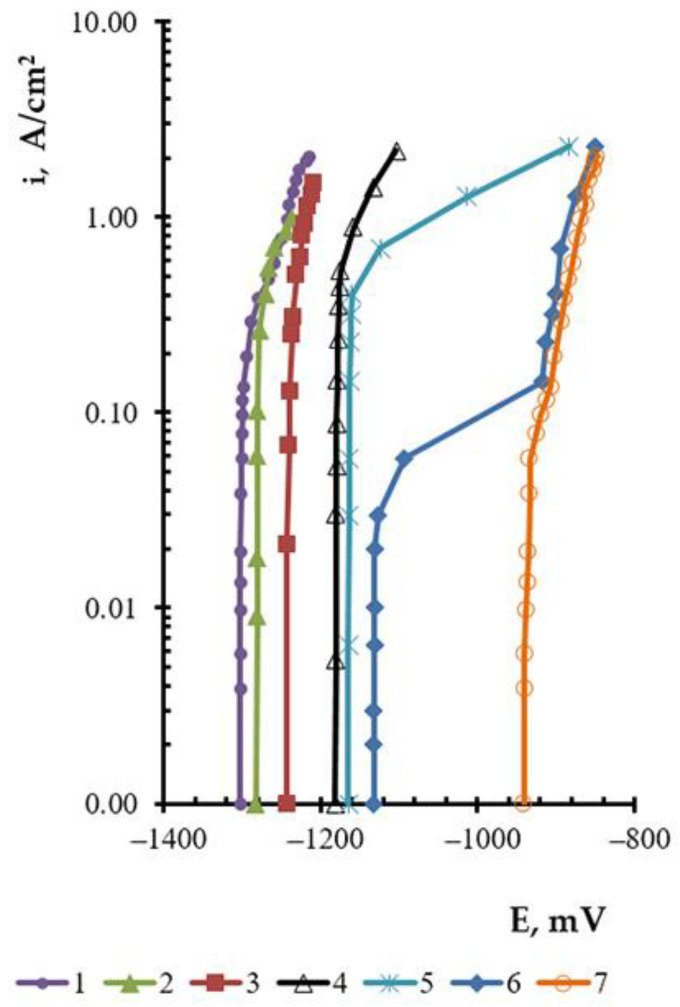
Experimentally obtained anode polarization of individual 3 Pb, Bi and double alloys at 823 K, mol.%: 1—Pb; 2—Pb–Bi (59.3–40.7); 3—Pb–Bi (32.5–67.5); 4—Pb–Bi (7.0–93.0); 5—Pb–Bi (5.0–95.0); 6—Pb–Bi (3.0–97.0); 7—Bi.

Figure 1 presents the measured anode polarization for binary alloys and metallic Pb and bismuth in the molten KCl–PbCl_2_ system.

**Figure 2:**
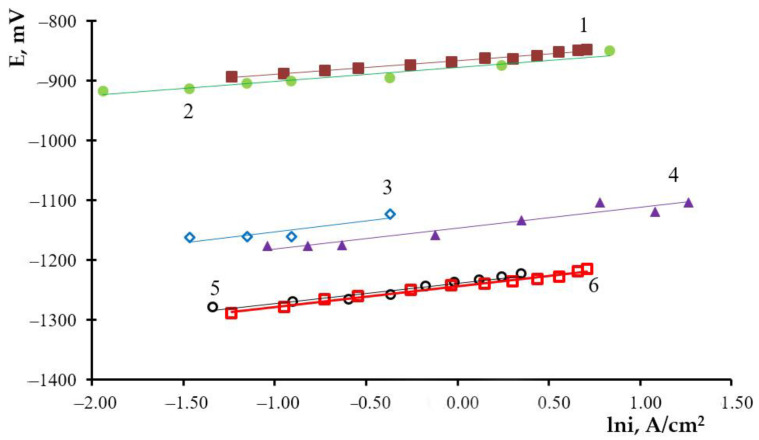
Straight line regions of lead, bismuth and Pb–Bi alloys polarization in the KCl–PbCl_2_ (50–50 mol.%) melt at 773 K, mol.%: 1—Bi; 2—Pb–Bi (3.0–97.0); 3—Pb–Bi (5.0–95.0); 4—Pb–Bi (7.0–93.0); 5—Pb–Bi (59.3–40.7); 6—Pb.

The general tendency towards changes in the anode polarization of double alloys, as the concentration of the most electronegative component decreases, is analogous to those observed for the liquid-metallic systems. A small lead concentration in the alloy has a substantial impact on the anode polarization. The illustrated curves of double alloys are located between those of metallic lead and metallic bismuth. As the anode current density changes from 0.001 to 0.03 A/cm^2^, the anode potential becomes constant. Within the current densities of 0.03 to 2.0 A/cm^2^, it shifts noticeably toward the electropositive values’ area. Curves 2–4 (Figure 1) illustrate that the anode potential under the experimental conditions does not reach the potentials of bismuth dissolution. It may be reached at the anode current densities of 2 A/cm^2^ (Figure 1, curve 5) and 0.1 A/cm^2^ (Figure 1, curve 5) for Pb–Bi (5.0–95.0) and Pb–Bi (3.0–97.0). The dissolution of double metallic Pb–Bi systems may be assumed to proceed in a diffusion regime. Within the current densities, corresponding to a distinguishable shift of the potential towards more positive values, a direct proportion between the potential and the current density logarithm is observed (Figure 2), as is the case of individual metals.

The equations of line and amount of electrons participation in the anode process both for individual metals and for double alloys are illustrated in Table 2.

As seen from Table 2, on average, two electrons participate in the anode reaction in liquid-metallic systems (3–5) and metallic lead (6) at noticeable deviations of potentials from equilibrium. This fact testifies that under given conditions, the ionization of lead follows the two-electron scheme and that double-charged ions form simultaneously in the electrolyte. When considering the alloys containing 5.7 and 59.3 mol.% of lead, the selective lead dissolution proceeds as follows:

Pb → Pb^2+^ + 2e^−^. (2)

The anode polarization for the Pb–Bi (5.0–95.0) and Pb–Bi (3.0–97.0) alloys increases, and anode potentials shift faster to the electropositive area. The lead ionization from double alloys proceeds under the limiting diffusion current. Only bismuth atoms remain at the anode layer surface at the above-mentioned current densities. Due to the fact that bismuth is the second electronegative component of the double alloy, the polarization curve repeats the run of the curve for the individual bismuth (curves 4 and 5, Figure 1).

The prior-to-logarithmic coefficient for individual bismuth and the Pb–Bi (3.0–97.0) alloy in the presented equations (1 and 2 in Table 2) is close to the value of R·T/3·n from the Nernst equation. Therefore, the alloy component ionization in this particular region of the polarization curve has a three-electron scheme and results in the formation of trivalent ions. The curve of the Pb–Bi alloy is located between polarization curves of lead and bismuth; under these conditions, selective bismuth dissolution takes place by the three-electron electrode reaction:

Bi→ Bi^3+^ + 3e^−^. (3)

To evaluate the diffusion layer thickness in the liquid-metal anode, it is favorable to use the value of the limiting diffusion current of metals [26]:

(4) $$\delta_{\mathrm{Pb}-\mathrm{Bi}}=\frac{{\mathrm{nFDC}}_{\mathrm{Me}}}{i_{\mathrm{Me}}}$$

where $\delta_{\mathrm{Pb}-\mathrm{Bi}}$ is the diffusion layer thickness in the alloy;

n is the number of electrons;

D is the diffusion coefficient of metallic atoms in the alloy;

C_Me_ is the concentration of the potential-determining component in the alloy;

i_Me_ is the limiting current density of metallic atoms diffusion from the alloy.

The value of lead diffusion coefficient D_Pb_ = 4.07 × 10^−5^ cm^2^/s in the alloy at the temperature of 823 K is taken from the Reference book [27]. The calculated values of diffusion layer thickness are presented in Table 3.

The diffusion layer thickness in the liquid lead–bismuth alloy for lead diffusing to the surface of the boundary between the alloy and the electrolyte is close to the value of the diffusion layer thickness in the molten system without mechanic stirring [28]. The presence of the clearly observed areas of the limiting diffusion current of electronegative component ionization in the region of small concentrations elucidates that the dissolution of individual elements from the alloy is highly selective. The decrease in the concentration of the electroactive component in the alloy results in the reduction of the limiting current of the component dissolution. Therefore, the degree of separation between lead and bismuth may be controlled by the limiting current of the electronegative component ionization.

**Figure 3:**
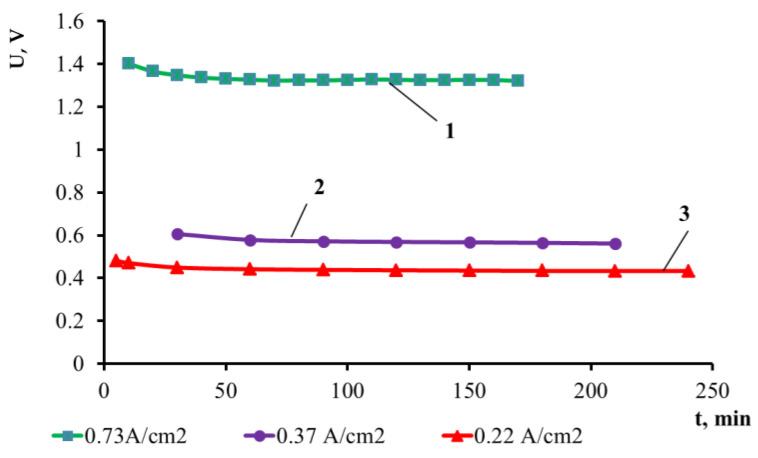
Time dependence of the electrodes’ voltage at the cathode current density: 1—0.73 A/cm^2^, 2—0.37 A/cm^2^, 3—0.22 A/cm^2^.

The extraction of lead from double alloys was performed by lead electrolysis [29] in the electrochemical cell for the thin-layer electrolytic refining of lead [23]. The porous ceramic diaphragm is a peculiarity of this cell. It has a form of a crucible prepared by the plasma spraying of the corundum ceramic powder with the desired value of the volume porosity and pore size. The volume porosity of the applied diaphragm is 30%, with the pore size being 10–30 µm. The ceramic material is permeable for the molten salt electrolyte and completely impermeable for the anode alloy and cathode lead. This porous ceramic diaphragm allows for the development of the construction of the electrochemical cell with the vertically located liquid-metal electrodes. Cathode lead is obtained inside the diaphragm, and bismuth alloy is loaded in the crucible outside the diaphragm. The porous diaphragm filled with the chloride KCl–PbCl_2_ electrolyte is located in the cell in such a way that the liquid-metal anode is separated from the cathode. Such design provides a chosen electrode spacing, which is significantly smaller than in the electrolytic cells of the “crucible-crucible” type [23]. Indeed, it is equal to the thickness of the diaphragm wall. Such design decreases the specific consumption of electric energy due to the decrease in the voltage between the electrodes. Figure 3 presents the function of the electrodes’ voltage, U and current density.

At the temperature of 823 K, the electrodes’ voltage of 0.48, 0.6 and 1.35 V was observed for the following values of the current densities: 0.22, 0.37 and 0.73 A/cm^2^, respectively. 

The electrolytic cells with porous ceramic diaphragms were tested under the following operating process parameters:
initial anode current density, A/cm^2^…………………….0.73initial cathode current density, A/cm^2^…………………..0.73process temperature, K …………………………………823final anode current density, A/cm^2^……………………..0.22final cathode current density, A/cm^2^……………………0.22KCl–PbCl_2_ electrolyte composition, mol.% …………...50:50

**Figure 4:**
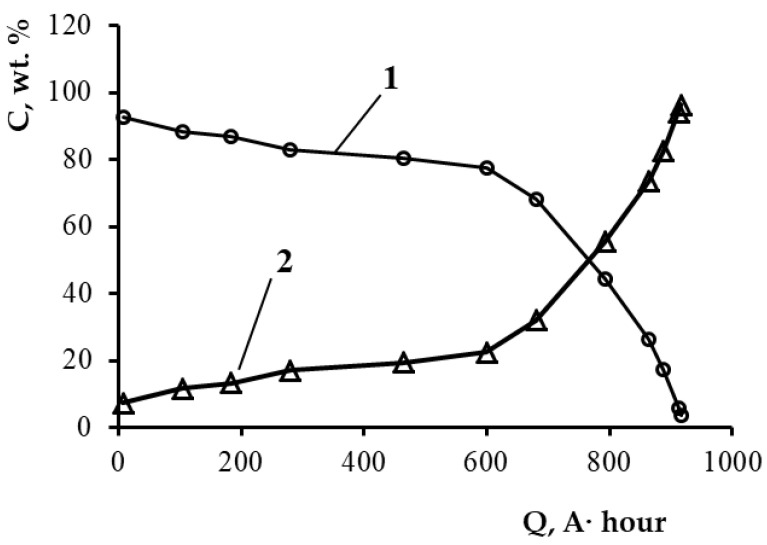
Dependence of the concentration of anode alloys components on the amount of electric charge passed: 1—Pb; 2—Bi.

Figure 4 presents the changes in the components’ concentration of the Pb–Bi alloy depending on the amount of electric charge, Q, passed during the electrolysis.

The electrochemical production of bismuth was performed under galvanostatic mode with the stepwise decrease in the current density. The first electrolysis stage included the decrease in the lead concentration in the anode alloy from 7 wt.% (Pb–Bi (93.0–7.0)) to 25 wt.% (Pb–Bi (75.0–25.0)) at the current density of 0.73 A/cm^2^. During the second electrolysis stage, the current density decreased further to 0.37 A/cm^2^; the process continued until the moment when the lead concentration in the anode alloy reached 12–15 wt.%. At the third electrolysis stage, the current density decreased to 0.22 A/cm^2^; the electrolysis terminated at the near-complete lead extraction from the anode alloy. Therefore, at the end of the process, the composition of the anode was as follows: Pb (3.5 wt.%)–Bi(96.5 wt.%). Selivanov et al. [30] report that the electrochemical dissolution of the alloy containing 8.72 wt.% of bismuth in the NaCl–KCl–PbCl_2_–ZnCl_2_ melt allowed them to obtain the final anode product containing 93 wt.% of bismuth. The technology described in the paper provides a twice smaller concentration of lead in the final anode alloy.

During the electrolysis, bismuth accumulates in the anode, whereas lead dissolves from the double alloy as the most electronegative component according to reaction (1). At the same time, Pb^2+^ ions reduce to Pb metal at the cathode. Therefore, lead is selectively transferred from the liquid-metallic anode to the cathode. As a result, the purity of the cathode lead is 99.99 wt.%.

## 4. Conclusions

The process of Pb–Bi alloys dissolution at the anode was studied in the molten KCl–PbCl_2_ system at 823 K at the current densities ranging from 0.001 to 2 A/cm^2^ at different concentrations of components in the alloys.

The analysis of the polarization curves elucidates that the alloys dissolve under diffusion mode. The transfer of the most electronegative component from the alloy bulk to the anode surface is the limiting stage. The number of bismuth and lead electrons participating in the electrode reactions is calculated according to the analytic description of the polarization curves. The anode dissolution of the Pb–Bi (59.3–40.7), Pb–Bi (32.5–67.5) and Pb–Bi (7.0–93.0) alloys proceeds by the two-electron reaction in the studied range of anode current densities. Therefore, the sole lead is transported to the melt in the form of Pb^2+^, whereas bismuth remains in the metallic alloy. The increase in the bismuth concentration in the alloy causes the change in the anode dissolution mechanism. That is why the Pb–Bi (5.0–95.0) and Pb–Bi (3.0–97.0) alloys dissolve electrochemically according to the three-electron reaction, i.e., bismuth is transported to the melt in the form of Bi^3+^ ions. The diffusion layer thickness in the liquid-metallic anode is calculated using the value of the metal limiting diffusion current. The diffusion layer thickness of the diffusing lead in the liquid Pb–Bi alloy is 0.01 cm, which is close to the value of the diffusion layer in the molten salt without mechanic stirring. The technology of the electrochemical bismuth production from the double alloys in the chloride mixture was tested in a laboratory-scale electrochemical cell. The electrolysis of the binary alloy of the Pb–Bi (93.0–7.0) composition in the electrochemical cell with a porous ceramic diaphragm applying three anode current densities of 0.73 A/cm^2^, 0.37 A/cm^2^ and 0.22 A/cm^2^ results in the formation of the Pb–Bi (3.5–96.5) alloy. This fact proves the possibility of electrochemical production of bismuth with small lead content.

## 5. Patents

A.P.A., K.Y.R., Z.Y.P., K.A.S., K.S.A., K.A.A., T.K.L. Method for electrolytic production of bismuth. Patent RU 2748451 C1 dated 30 November 2020.

## Figures and Tables

**Table 1:** Working electrodes’ compositions (mol.%).

No.	Bi	Pb
1	0.0	100.0
2	32.5	67.5
3	59.3	40.7
4	93.0	7.0
5	95.0	5.0
6	97.0	3.0
7	100.0	0.0

**Table 2:** Equations of lines and number of electrons, calculated according to the angular coefficient at 823 K.

No.	Compositon, mol.%	Equation E, V	R^2^	n
1	Bi	E = −0.8665 + 0.0231·lni_a_	0.98	3.07
2	Pb–Bi (3.0–97.0)	E = −0.8775 + 0.0237·lni_a_	0.95	2.99
3	Pb–Bi (5.0–95.0)	E = −1.1168 + 0.0358·lni_a_	0.79	1.98
4	Pb–Bi (7.0–93.0)	E = −1.1470 + 0.0348·lni_a_	0.93	2.02
5	Pb–Bi (59.3–40.7)	E = −1.2386 + 0.0343·lni_a_	0.95	2.06
6	Pb	E = −1.2432 + 0.0348·lni_a_	0.99	2.03

**Table 3:** The thickness of the diffusion layer in liquid alloys at 823 K.

Diffusing Element	Alloy Composition, mol/%	C_Me_, mol/cm^3^	i_Me_, A/cm^2^	δ, cm
Pb	Pb–Bi (5.0–95.0)	0.0008	0.7	0.01

## Data Availability

Not applicable.
